# It’s Not Just a Yes or No Answer: Expressions of Local Health Department Accreditation

**DOI:** 10.3389/fpubh.2016.00021

**Published:** 2016-02-16

**Authors:** Beth E. Meyerson, Jerry King, Karen Comer, Sandra S. Liu, Laura Miller

**Affiliations:** ^1^Department of Applied Health Science, Indiana University School of Public Health-Bloomington, Bloomington, IN, USA; ^2^Indiana Public Health Association, Indianapolis, IN, USA; ^3^The Polis Center, Indiana University–Purdue University at Indianapolis, Indianapolis, IN, USA; ^4^College of Health and Human Sciences, Purdue University, West Lafayette, IN, USA

**Keywords:** local health departments, public health accreditation, performance improvement, public health organizational development, public health administration

## Abstract

The identification and exploration of moderators of health department accreditation remain limited by current dichotomous conceptualizations of pursuit.

**Methods:**

A 2015 survey measured Indiana local health department (LHD) accreditation pursuit and progress, classifying respondents by progress evidence. Covariates included attitudes about the future impact of accreditation on funding and performance, health department size, geography, health outcome ranking, and quality improvement (QI) programing.

**Results:**

Four classifications of accreditation pursuit emerged and were found to have greater association with covariates than standard dichotomous measures. “Active Pursuit” was associated with formal QI programing and a belief that accreditation will impact future funding and performance. “Intent Only” was associated with no QI programing and no completion of accreditation prerequisites. “Discontinued” was associated with the belief that accreditation will not impact future performance. “Not Pursuing” was associated with no interest or plan to complete prerequisites and reported belief that accreditation will not impact future health department funding or performance.

**Conclusion:**

More granular characterizations of accreditation pursuit may improve understanding of influential factors. This measurement framework should be validated in studies of LHDs in other states.

## Introduction

U.S. health department pursuit of voluntary accreditation is a natural experiment allowing the observation, identification, and definition of influential environmental, organizational, policy, and structural factors. The national research agenda set by the Public Health Accreditation Board’s (PHAB) Research Advisory Council called for improved measures to help understand this pursuit and progress (1). While local health department (LHD) accreditation is recognized as a QI initiative that could help standardize the delivery of 10 essential public health services in each community (2–4), it is a fairly new innovation, launched nationally in 2011.

The study of accreditation has quickly advanced as much as this evolving implementation environment will allow. It has become clear that financial and legal incentives facilitate accreditation, as do population size, degree of top executive, governance structure, state health department accreditation pursuit, and the existence of formal QI initiatives (5–8). The influence of prerequisite completion continues to be investigated (5, 9, 10), and frameworks guiding the study of accreditation are at formative stage; two have used the PHAB framework for accreditation to help conceptualize progress (5, 11).

Local health department accreditation adoption is likely more nuanced than being in a state of complete pursuit or non-pursuit and might be better characterized as periods of starting and stopping, with perhaps conflicting decisions about whether to continue pursuit. The influence of factors found to associate with accreditation pursuit and progress may be more pronounced when adoption is conceptualized in more than dichotomous terms such as “yes, we are pursuing” or “no, we are not pursuing.”

Therefore, we think that accreditation pursuit and non-pursuit can be further characterized to advance our understanding of influencing factors and to inform the development of structural interventions that may facilitate accreditation pursuit. In practice, we have observed LHDs dropping out of the accreditation process at a variety of points along the way; however, our prior attempt at “staging” these moments was unsuccessful (11). Greater clarity of accreditation pursuit and progress in terms of its permanency, stability, or tenuousness would inform the development of frameworks to guide further inquiry.

We hypothesize that a more “granular” conceptualization of accreditation pursuit and progress would advance our understanding of influencers. We also hypothesize that beliefs about the future impact of accreditation on funding and performance may also moderate leadership decisions to pursue accreditation. This study seeks to classify expressions of accreditation pursuit with the purpose of discerning factors influencing pursuit and progress. This also involved the preliminary investigation of attitudes about the future impact of accreditation.

Indiana’s 92 counties are served by 93 LHDs. Two health departments (East Chicago and Gary) are city health departments, one health department (Fountain/Warren) is a two-county health department, and the remaining 90 are county health departments. All Indiana LHDs are units of the local government. The 2014 per capita state public health expenditure was $13.08 (down from $17.43 in 2013), placing Indiana 44th in the US for state public health investment (12). Indiana is characterized as 53.7% rural, with 27.6% of the population living in those rural areas (13). A majority of counties (70.7%) have populations under 50,000, 27.2% have populations under 25,000, and 3.3% have populations under 10,000. This is similar to the distribution of health departments nationally, for the exception of counties serving populations under 50,000. According to the 2013 National Association of County and City Health Officials Profile of Local Health Departments, 61% of U.S. local health departments serve counties under 50,000 (14).

There are no regulations or laws in Indiana with regard to accreditation, though there is a statewide Indiana Accreditation Partnership convened by the Indiana Public Health Association (IPHA) and including governmental, non-governmental, and academic partners focused on advancing accreditation. To date, neither the Indiana State Department of Health nor any Indiana LHD has achieved PHAB accreditation.

## Materials and Methods

### Measures

This study is a cross-sectional observational study to classify LHD expressions of accreditation pursuit in Indiana and to test associations with selected covariates. An online 35-item survey with closed and open-ended questions was conducted by the IPHA and sent to all 93 LHD directors. The survey period was from November 2014 to March 2015.

An *a priori* conceptualization of likely accreditation pursuit classified survey responses into the following categories: accreditation pursuit (“yes”), not pursuit (“no”), and discontinuation of accreditation pursuit (“started accreditation pursuit, but no longer are pursuing”).

Accreditation progress was categorized using four of the seven PHAB steps to the accreditation process: pre-application, application, documentation, and site visit (15). The PHAB steps are essentially categories of organizational behaviors related to accreditation. As none of the Indiana health departments achieved accreditation to date, we used only those steps that were relevant to the sample. Response options for each subactivity related to pre-application included (a) not completed, (b) is currently preparing to initiate, (c) have initiated, and (d) are completed. Prerequisites of community health assessment, community health improvement plan, and strategic plan were also measured by level of completion as (a) current, (b) in midst of completion, (c) initiating next year, and (d) “does not have and will not complete in the near future.” Prerequisites were understood to be part of ongoing cyclical planning processes.

The following LHD characteristics were compared with accreditation pursuit and progress: LHD size as measured by population and classified using categories from the National Association of County and City Health Officials (NACCHO): 100,000–499,999, 500,000–999,999, and <1,000,000, and the PHAB, which split the smaller population category into two sizes: LHDs serving populations of <50,000 and LHDs serving populations of 50,000–99,999.

Other LHD characteristics of interest included county health outcome ranking for 2014 (16) and rurality as classified by the U.S. Department of Agriculture: metropolitan (counties in metropolitan areas), non-metropolitan (counties adjacent to metropolitan areas with populations ranging of ≥2,500), and completely rural (counties far from metropolitan areas and/or with <2,500 population) (17).

Finally, we measured the existence and extent of QI programing and LHD attitudes about the likely future impact of accreditation on funding and performance. QI programing was measured as (a) formal, agency-wide QI programing, (b) formal QI programing for a specific program or functional area, (c) informal or *ad hoc* QI activities, and (d) no current involvement with QI activities. Attitudes about the potential future impact of accreditation on funding and performance was measured using a five-point Likert scale of agreement with two statements: (1) *In the future, there will likely be a difference in funding levels between accredited and non-accredited health departments* and (2) *In the future, there will likely be a difference in performance between accredited and non-accredited health departments*.

### Data Analysis

Reported LHD accreditation pursuit, attitudes, and accreditation barriers were coded as dichotomous variables to allow bivariate comparison with LHD characteristics. Categorization of reported health department accreditation intent and reported activity informed the delineation of LHDs into distinct groups based on intent to pursue and evidence of accreditation pursuit. These groups became categories of LHD types that were compared with LHD characteristics. Bivariate comparisons were tested using Chi-square goodness-of-fit and reported at the *p* < 0.5 level.

Open-ended questions were used to gather explanatory rationale for decisions to pursue accreditation, or perceptions of factors influencing accreditation decision making and pursuit. Qualitative data from these questions were coded thematically and analyzed by predominant theme. The study was deemed exempt by the Indiana University Institutional Review Board.

## Results

### Sample

The response rate was 74.2%, with 69 of the 93 Indiana LHDs. These health departments reflected their peers across the state in terms of population size, rurality, and county health outcome ranking. A majority of the sample (62.3%) served county populations of <50,000, 21.7% served populations of 100,000–499,999, 14.5% served populations of 50,000–99,999, and 1.4% served populations ≥500,000. Metropolitan areas with multiple counties and high social and economic integration were served by 56.5% of LHDs, and 2.9% of LHDs served completely rural populations that were not adjacent to metropolitan areas.

A majority of LHDs reported some form of QI programing, as 42% reported implementing informal or *ad hoc* QI activities, 13% reporting formal, program-specific QI programing, and 11.6% reporting formal, agency-wide-specific QI programing.

Over half of the LHDs (52.2%) agreed or strongly agreed that there would likely be funding differences between accredited and non-accredited health departments in the future, and most responded with agreement or neutrality to both funding and performance impact questions. Those who strongly agreed that there would likely be funding differences between accredited and non-accredited health departments also tended to strongly agree that there would likely be performance differences (*X*^2^ = 20.4, FET *p* = 0.000). This was also observed among those who expressed agreement (*X*^2^ = 10.4, FET *p* = 0.002), neutrality (*X*^2^ = 9.9, *p* = 0.002), disagreement (*X*^2^ = 7.9, FET *p* = 0.024), and strong disagreement (*X*^2^ = 37.2, FET *p* = 0.000).

#### Accreditation Pursuit and Progress

Over half (60.9%, 42) of the 69 responding LHDs reported pursuit of accreditation. These health departments reflected the distribution of the entire sample across characteristics measured. When understood dichotomously as “yes” pursuing and “no” not pursuing, we observed there to be no statistically significant relationships between accreditation pursuit and LHD population size, metropolitan designation, or health outcome ranking. However, reported accreditation pursuit was associated with beliefs about future impact on funding and performance. Those pursuing accreditation were four times more likely to agree or strongly agree that accreditation would have a future impact on LHD funding (*X*^2^ = 7.6, *p* = 0.006; OR: 4.2, CI: 1.5–11.7) and 28 times more likely to agree or strongly agree that it would affect performance (*X*^2^ = 17.4, *p* = 0.000; OR: 28.7, CI: 3.5–232.7). Notably, while those LHDs who pursued accreditation believed in the future impact of accreditation on funding and performance, the converse was not true: LHDs who believed in the future impact of accreditation on funding and performance did not necessarily report pursuing accreditation. In contrast to national findings, formal agency-wide and program/focal area QI programing was not associated with the reported pursuit of accreditation.

Progress toward accreditation was measured using reported behaviors associated with four of PHAB’s Seven Steps of Accreditation measures (see Table 1).

**Table 1:** **Reported accreditation progress by Indiana local health departments, according to PHAB accreditation steps 2015 (*N* = 42)**.

		No activity *N* (%)	Preparing to initiate *N* (%)	Initiated *N* (%)	Completed *N* (%)
Pre-application	Review information or attend training about national, voluntary public health accreditation from PHAB	**19 (45.2)**	4 (9.5)	7 (16.7)	12 (28.6)
	Self-assessment against PHAB Standards Measures	**20 (47.6)**	8 (19.0)	7 (16.7)	7 (16.7)
	Identify strengths and weaknesses based on self-assessment	**21 (50.0)**	11 (26.2)	5 (11.9)	5 (11.9)
	Address strengths and weaknesses	**23 (54.8)**	12 (28.6)	4 (9.5)	3 (7.1)
	Complete readiness checklist	**30 (71.4)**	3 (7.1)	4 (9.5)	5 (11.9)
	Complete PHAB orientation	**32 (76.2)**	3 (7.1)	1 (2.4)	6 (14.3)
	Submit PHAB Statement of Intent	**37 (88.1)**	4 (9.5)	–	1 (2.4)
Application	Complete and submit application to PHAB	**38 (90.5)**	3 (7.1)	–	1 (2.4)
	Send application fee to PHAB	**39 (92.9)**	2 (4.8)	–	1 (2.4)
	Complete PHAB training (post-application)	**40 (95.2)**	1 (2.4)	–	1 (2.4)
Documentation selection and submission	Submit documentation to PHAB	**40 (95.2)**	1 (2.4)	–	1 (2.4)
Site visit	PHAB site visit	**39 (92.9)**	1 (2.4)	1 (2.4)	1 (2.4)
	Receive and review PHAB site visit report	**40 (95.2)**	1 (2.4)	–	1 (2.4)

*Emphasis (bold) noting health departments reporting accreditation pursuit without evidence of progress*.

As shown in Table 1, 25 (59.5%) LHDs pursuing accreditation were beginning or in the midst of the pre-application stage, as they indicated that they were “preparing to initiate” or they “initiated” one or more of the activities in this step. None had initiated application activities, though it appears that four (9.5%) LHDs are almost to this point, as they reported preparing to initiate their PHAB statement of intent. Another two LHDs (4.8%) were at the site visit step. There were no statistically significant associations with progress by step and LHD characteristics. Reported activities at each accreditation step were also compared with health department characteristics of size and metropolitan designation. Here again, there were no statistically significant associations found.

Notably, several health departments reported no activity on several PHAB application steps and associated behaviors, despite reporting intent to pursue accreditation (see bold text in Table 1). Of this group, 15 (35.7%) reported no activity on *any progress indicators*. This observation led to the separation of LHDs pursuing accreditation into two groups: those 15 LHDs who indicated intent but reported no evidence of pursuit were categorized as “Intent Only.” The 27 (64.3%) LHDs who indicated accreditation intent and reported evidence of pursuit were categorized as “Active Pursuit.”

Examination of prerequisite completion provided another opportunity to assess whether “Intent Only” LHDs were at least pursuing prerequisites to accreditation, even though completion of these preparation activities had not been found to predict accreditation pursuit (see Table 2).

**Table 2:** **Reported prerequisite completion for Indiana LHDs pursuing accreditation by accreditation progress classification, 2015 (*N* = 42)**.

	Intent Only (*N* **=** 15)	Active Pursuit (*N* **=** 27)	Total (*N* **=** 42)
**Community health assessment “CHA”**			
Is current	5 (33.3)	13 (48.1)	18 (42.9)
In midst of completing	–	6 (22.2)	1 (14.3)
Initiating next year	3 (20.0)	8 (29.6)	11 (26.2)
Does not have and will not complete in near future	4 (26.7)	–	4 (9.5)
Did not answer, presumed no activity	3 (20.0)	–	3 (7.1)
**Community health improvement plan “CHIP”**			
Is current	2 (13.3)	7 (25.9)	9 (21.4)
In midst of completing	–	3 (11.1)	3 (7.1)
Initiating next year	2 (13.3)	15 (55.6)	17 (40.5)
Does not have and will not complete in near future	7 (46.7)	2 (7.4)	9 (21.4)
Did not answer, presumed no activity	4 (26.7)	–	4 (9.5)
**Strategic plan**			
Is current	2 (13.3)	10 (37.0)	12 (28.6)
In midst of completing	1 (6.7)	7 (25.9)	8 (19.0)
Initiating next year	2 (13.3)	8 (29.6)	10 (23.8)
Does not have and will not complete in near future	5 (33.3)	2 (7.4)	7 (16.7)
Did not answer, presumed no activity	5 (33.3)	–	5 (11.9)

For those 15 LHDs who reported intent to pursue accreditation but did not report evidence of accreditation progress, almost half (46.7%, 7) reported not having a community health assessment and did not have plans to complete one in the near future. A majority (73.3%, 11) reported the same outcome for the community health improvement plan, and 10 (66.6%) indicated the same decision for a strategic plan.

Prerequisites completion was associated with beliefs about the future impact of accreditation on LHD funding and performance and with reported QI programing. LHDs reporting the completion of at least one prerequisite (CHA, CHIP, and/or strategic plan) tended to agree or strongly agree that accreditation would have a future impact on funding (*X*^2^ = 9.9, *p* = 0.002; OR: 7.5, CI: 1.9–29.1) and performance (*X*^2^ = 7.6, *p* = 0.008; OR: 4.6, CI: 1.5–14.3). LHDs reporting formal agency-wide or program-specific QI programing tended to report that all prerequisites were complete (*X*^2^ = 5.3, *p* = 0.04; OR: 5.0, CI: 1.2–21.5). Conversely, LHDs reporting that they were not engaged in any of the prerequisites and did not plan to be in the near future tended to disagree or strongly disagree that accreditation would impact future LHD performance (*X*^2^ = 12.6, *p* = 0.001; OR: 8.8, CI: 2.4–35.5).

While associations between formal QI programing and accreditation pursuit were not observed for the 42 LHDs reporting pursuit of accreditation, associations were observed when further distinguishing reported accreditation pursuit by progress evidence. For example, LHDs reporting intent to pursue accreditation and reporting measurable progress toward it (“Active Pursuit”) tended to report formal agency-wide QI programing (*X*^2^ = 8.9, *p* = 0.005; OR: 14.4, CI: 1.7–124.7). Conversely, the “Intent Only” LHDs were unlikely to report completing one or more of the prerequisites (*X*^2^ = 3.6, *p* = 0.05; OR: 0.281, CI: 0.07–1.1) and likely to report preference not to engage in the prerequisites of community assessment, community health improvement planning, and strategic planning (*X*^2^ = 6.1, *p* = 0.02; OR: 4.2, CI: 1.3–14.2).

#### Not and No Longer Pursuing Accreditation

Twenty-seven (39.1%) of 69 responding LHDs indicated that they were not pursuing accreditation. Of this group, over half (51.9%, 14) reported not ever pursuing accreditation and 13 (48.1%) reported discontinuing accreditation pursuit after having started it. These two expressions of accreditation pursuit (or non-pursuit) were classified as “Not Pursuing” and as “Discontinued” pursuit.

Local health departments reporting never having pursued accreditation tended to disagree or strongly disagree that accreditation would impact the future LHD performance (*X*^2^ = 12.6, *p* = 0.001; OR: 8.8, CI: 2.4–35.2). They also tended *not to* agree or strongly agree that accreditation would impact future LHD funding (*X*^2^ = 7.4, *p* = 0.007; OR: 4.2, CI: 1.3–13.9). These LHDs were generally not engaged or did not plan to engage in the near future, in prerequisite activities of community health assessment, community health improvement planning, or strategic planning (*X*^2^ = 8.2, *p* = 0.009; OR: 5.9, CI: 1.6–21.3).

The 13 LHDs no longer pursuing accreditation (“Discontinued”) spent between <1 year and 5 years considering or working on accreditation (SD: 1.7). Most (76.9%, 10) reported having spent an average of 2.2 years considering or working on accreditation activities with 4 (40%) spending 2 years, 2 (20%) spending 4 years, and 2 (20%) deciding not to pursue accreditation during the same year they began (both initiating in 2012). One health department spent 1 year working on or considering accreditation (from 2013 to 2014), and the remaining health department reported spending 5 years because they began accreditation consideration or pursuit in 2010 and indicated that they had “*not totally stopped, just postponing aggressive efforts but continuing to gather information and weigh our options*.”

Notably, the only association observed for LHDs who discontinued accreditation pursuit was that they tended *not to agree or strongly agree* that accreditation would impact future LHD performance (*X*^2^ = 5.3, *p* = 0.02; OR: 6.3, CI: 0.89–45.5). Of these 13 LHDs, 3 explained that their decision was based on the need to focus on other priorities, such as seeking status as a Federally Qualified Health Center, or that the “*timing was not right”* for accreditation. All three indicated that they might elect to pursue accreditation at some point in the future. Four of seven small health departments (<50,000 population) in this category indicated discontinuing accreditation pursuit because the application fees were too high, and five indicated that the time and effort required exceeded the capacity of their health department.

## Discussion

As our findings indicated, when observing accreditation pursuit only in dichotomous terms “yes/no,” there was little association between accreditation pursuit and LHD characteristics. Therefore, this approach limited our understanding of factors influencing accreditation pursuit and progress. That associations were not found between LHDs reporting “yes” to accreditation pursuit and LHD behavior, population, or attitude characteristics, yet observed when distinguishing LHDs by more granular understandings of their pursuit and progress, suggests that such framing may be valuable when trying to understand accreditation pursuit over time.

**Figure 1:**
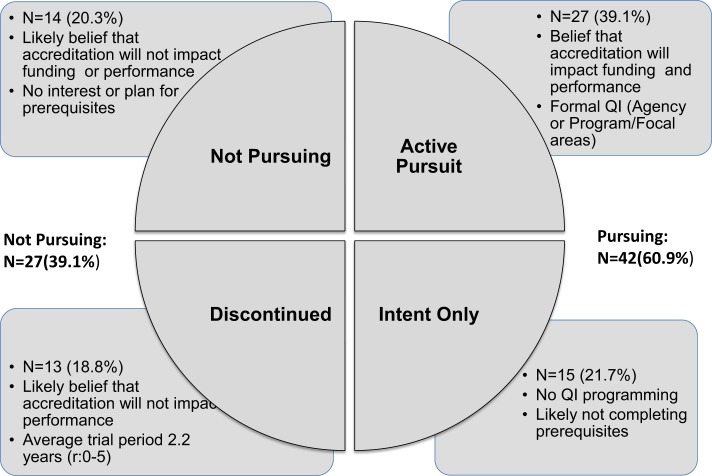
**Emerging categories of local health accreditation adoption, Indiana Survey 2015 (*N* = 69)**.

More was yielded when we attempted to classify accreditation pursuit in terms of documented progress using the PHAB framework and when creating a classification for discontinuation. What emerges is a picture of four types of health department pursuit of accreditation (Figure 1).

Understanding accreditation pursuit and progress in this way leads to questions about directionality. For example, the feedback from LHDs who initiated and then discontinued accreditation suggests that decisions to discontinue might change over time. Thus, it might be more helpful to understand LHDs discontinuing accreditation pursuit as being in a process of organizational evolution that may lead to discernable accreditation progress over time. Some of these LHDs expressed intention to resume, while others did not. These observations might help to discern a moderating factor of leadership beliefs about accreditation impact on funding and performance. That said, while accreditation in public health as well as other fields is seen as a quality-initiative, public health accreditation is not necessarily a uniformly shared goal across the country. Our intent here is not to suggest that accreditation should be sought by all health departments. It is, instead, to understand accreditation adoption as structural effort to advance public health performance improvement.

Using this framework to understand accreditation among a larger number of LHDs across the country would advance our current accreditation literature because there is now more than two categories of accreditation pursuit. Findings might also yield greater understanding of what accreditation pursuit looks like over time and what characteristics or elements might facilitate diagonal or lateral movement around adoption expressions as shown in Figure 1. As LHDs continue to adopt accreditation across the country, lateral movement from “Not Pursuing” to “Active Pursuit” would likely become the biggest leap requiring the largest intervention. Given limited accreditation resources, those engaged in facilitating accreditation pursuit would need to prioritize LHDs for technical assistance resources, but on what basis? Which characteristics would serve as key moderators for accreditation progress? Experience over time will likely show a decision by LHDs to drop out of the process altogether, suggesting a fifth category for the framework such as “Permanent Discontinuation.” But how permanent is this decision with leadership change at the LHD level? What is the impact of such a classification label on potential future organizational behavior?

Our study was the first to introduce the concept of attitudes about accreditation and its association with pursuit and progress. This may be particularly helpful when understanding the four LHD accreditation pursuit categories over time, as the belief attribute might play a more central role in leadership decisions of accreditation pursuit or pacing. While we did not seek to understand whether belief facilitates (precedes) or reinforces (follows) accreditation pursuit, we can see distinct differences in beliefs among LHDs based on where they are pursuing accreditation. Those who are not pursuing accreditation, whether “delaying pursuit” or “not yet pursuing,” held beliefs about the impact of accreditation, which might serve to reinforce behavior in some important ways. Further and deeper exploration of accreditation discontinuation or de-adoption is warranted.

Indiana, like many states, lacks structural facilitators of accreditation such as system-wide accreditation funding, legal tools (18), or public health law as in Ohio, where local health districts are required to achieve accreditation by 2020 (19). Therefore, understanding accreditation pursuit and progress differentiation will allow more meaningful understanding of indicators and their influence of this performance improvement initiative over time.

Study limitations included setting and study design. The limitation of setting involved the fact that Indiana has a preponderance of counties with populations of <50,000. Exploring the relevance of this framework across several states will further the understanding of the impact of population size as a potential covariate across potential expressions of accreditation intent and pursuit. The limitation of study design involved the use of only four of seven PHAB steps based on the current accreditation progress in Indiana. Studies in states with accredited health departments would help to validate the adoption framework emerging from this study. Finally, data were gathered through self-reported survey responses, and as such, there were no efforts to validate what was reported.

## Author Contributions

BEM conceived of and directed all aspects of the study and the manuscript preparation. JK participated in the study planning and supported implementation. He interpreted the findings and participated in the manuscript editing. KC assisted the development of the survey instrument and edited the manuscript. SL gathered and interpreted data, and edited the manuscript. LM gathered data and edited the manuscript.

## Conflict of Interest Statement

BM does not have anything to disclose for this study and manuscript. Corporate relationships are as follows: BM has received research funding from Roche Diagnostics and from GlaxoSmithKline for studies in cervical cancer policy. She is also affiliated with the Indiana University School of Medicine, the Kinsey Institute, and the Center for HPV Research. JK, KC, SL, and LM do not have anything to disclose.

## References

[1] Riley WJ Lownik EM Scutchfield FD Mays GP Corso LC Beitsch LM Public health accreditation: setting the research agenda. Am J Prev Med (2012) 42(3):263–71. 10.1016/j.amepre.2011.10.021 22341163

[2] Robert Wood Johnson Foundation. Providing Assistance to Public Health Agencies for Accreditation Report. Princeton, NJ (2012).

[3] Brewer RA Joly M Mason M Tews D Thielen L Lessons learned from a multistate learning collaborative. J Public Health Manag Pract (2007) 13(4):388–94. 10.1097/01.PHH.0000278033.64443.2a 17563628

[4] Thielen L Exploring Public Health Experience with Standards and Accreditation. Is It Time to Stop Talking about How Every Health Department Is Unique? Report Prepared for the Robert Wood Johnson Foundation (2000). Available from: http://www.cdc.gov/nceh/ehs/ephli/resources/exploring_public_health.pdf

[5] Beatty KE Mayer J Elliott M Brownson RC Abdulloeva S Wojciehowski K Barriers and incentives to rural health department accreditation. J Public Health Manag Pract (2016) 22(2):138–48. 25867493 10.1097/PHH.0000000000000264

[6] Yeager VA Ye J Kronstadt J Robin N Leep C Beitsch LM National voluntary public health accreditation: are more local health departments intending to take part? J Public Health Manag Pract (2015). 10.1097/PHH.0000000000000242 25851799

[7] Yeager VA Ferdinand AO Beitsch LM Menachemi N. Local public health department characteristics associated with likelihood to participate in national accreditation. Am J Public Health (2015) 105:1653–9. 10.2105/AJPH.2014.302503 26066930 PMC4504315

[8] Shah GH Leep C Ye J Sellers K Liss-Levinson R Williams KS Public health agencies’ level of engagement in and perceived barriers to PHAB national voluntary accreditation. J Public Health Manag Pract (2015) 21(1):107–15. 10.1097/PHH.0000000000000117 25010327

[9] Shah GH Beatty K Leep C Do PHAB accreditation prerequisites predict local health departments’ intentions to seek voluntary national accreditation? Front Public Health Serv Syst Res (2013) 2(3):4.

[10] Beatty KE Mayer J Elliott M Brownson RC Wojciehowski K Patterns and predictors of local health department accreditation. J Public Health Manag Pract (2015) 21(1):116–25. 10.1097/PHH.0000000000000089 24722052 PMC4918814

[11] Meyerson BE Barnes PR King J Halverson P Degi L Polmanski HF Measuring accreditation activity and progress: findings from a survey of Indiana local health departments. Public Health Rep (2015) 130(5):447–52. 26327722 10.1177/003335491513000507 PMC4529828

[12] Trust for America’s Health. Investing in America’s Health: A State-By-State Look at Public Health Funding and Key Health Facts. Report. Washington, DC: TFAH (2015).

[13] U.S. Department of Commerce, United States Census Bureau. Percent Urban and Rural in 2010 by State and County [Online] (2015). Available from: https://www.census.gov/geo/reference/ua/urban-rural-2010.html

[14] National Association of County and City Health Officials. 2013 National Profile of Local Health Departments. Report. Washington, DC: NACCHO (2014).

[15] Public Health Accreditation Board. Guide to National Public Health Accreditation, Version 1.0 [Online] (2011). Available from: www.­phaboard.org

[16] University of Wisconsin Population Health Institute. County Health Rankings (2012). Available from: www.countyhealthrankings.org

[17] U.S. Department of Agriculture Economic Research Service. Rural-Urban Commuting Area Codes, 2010 [Online] (2015). Available from: http://www.ers.usda.gov/data-products/rural-urban-commuting-area-codes.aspx

[18] Thielen L Dauer E Burkhardt D Lampe S VanRaemdonck L An examination of state laws and policies regarding public health accreditation prerequisites. J Public Health Manag Pract (2014) 20(1):111–8. 10.1097/PHH.0b013e3182a505c9 24322704

[19] Ohio Revised code § 3701.98 Standards, Policies and Procedures. Available from: http://codes.ohio.gov/orc/3701.98

